# Evaluation of exposure dose in fetal computed tomography using organ-effective modulation

**DOI:** 10.1007/s13246-020-00921-z

**Published:** 2020-09-14

**Authors:** Masanao Kobayashi, Tomonobu Haba, Sayaka Suzuki, Yusei Nishihara, Yasuki Asada, Kazuyuki Minami

**Affiliations:** 1 Graduate School of Medical Sciences, Fujita Health University, 1-98 Dengakugakubo, Kutsukake-cho, Toyoake, Aichi Japan; 2 Department of Radiology, Fujita Health University Hospital, 1-98 Dengakugakubo, Kutsukake-cho, Toyoake, Aichi Japan

**Keywords:** Organ-effective modulation, Fetal CT scan, Organ exposure, Monte Carlo simulation

## Abstract

Organ-effective modulation (OEM) is a computed tomography scanning technique that reduces the exposure dose to organs at risk. Ultrasonography is commonly used for prenatal imaging, but its reliability is reported to be limited. Radiography and computed tomography (CT) are reliable but pose risk of radiation exposure to the pregnant woman and her fetus. Although there are many reports on the exposure dose associated with fetal CT scans, no reports exist on OEM use in fetal CT scans. We measured the basic characteristics of organ-effective modulation (X-ray output modulation angle, maximum X-ray output modulation rate, total X-ray output modulation rate, and noise modulation) and used them in a Monte Carlo simulation to evaluate the effect of this technique on fetal CT scans in terms of image quality and exposure dose to the pregnant woman and fetus. Using ImPACT MC software, Monte Carlo simulations of OEM_ON_ and OEM_OFF_ were run on 8 cases involving fetal CT scans. We confirmed that the organ-effective modulation X-ray output modulation angle was 160°; the X-ray output modulation rate increased with increasing tube current; and no modulation occurred at tube currents of 80 mA or below. Our findings suggest that OEM has only a minimal effect in reducing organ exposure in pregnant women; therefore, it should be used on the anterior side (OEM_ON,front_) to reduce the exposure dose to the fetus.

## Introduction

Ultrasonography (US) is commonly used for prenatal image-based diagnosis of pregnancy [1–3], although opinion is divided on this matter, as some reports state that the reliability of US is limited by acoustic impedance [4, 5]. Radiography and computed tomography (CT) both demonstrate good diagnostic performance for the bone, but they are transmission-based techniques requiring radiation exposure to both the fetus and the pregnant woman. The exposure dose received by the fetus in these radiation-based diagnostic techniques is under 100 mGy, and it almost never increases the risk of prenatal death, deformity, or mental retardation by an amount detectable above spontaneous incidence [6]. ICRP Publication 84 mentions that detailed informed consent is required when the fetal exposure dose exceeds 1 mGy and that methods of reducing exposure should be investigated when the fetus is exposed directly [6]. For these reasons, we should endeavor to reduce exposure through introduction of exposure-reducing techniques when using radiation in prenatal image-based diagnoses.

Organ-effective modulation (OEM) is a recent development in the field of CT system scanning techniques that restricts X-ray output over specific tube orientations to reduce the exposure dose to organs at risk, such as the lens of the eye and the breast [7–9]. Many reports have evaluated the exposure dose in fetal CT scans [10–12], but no reports have used OEM in fetal CT scans and investigated the effects in detail. Moreover, the American College of Radiology and the Society for Pediatric Radiology have recommended the use of auto-exposure control techniques but have not discussed the possibility of reducing patient exposure with the recently developed OEM technique [13].

Herein, we investigated the potential clinical application of OEM in fetal CT scans by measuring the basic characteristics of OEM (X-ray output modulation angle, maximum X-ray output modulation rate, total X-ray output modulation rate, and noise modulation) and using them to run a Monte Carlo (MC) simulation, thereby evaluating the effect of OEM on fetal CT scans in terms of image quality and exposure dose to the fetus and the pregnant woman.

## Methods

### Evaluating the basic characteristics of OEM

A 32-cm-diameter polymethyl methacrylate (PMMA) phantom was positioned with its center at the isocenter of the CT system (Aquilion ONE Vision Edition, Canon Medical Systems, Otawara, Japan). A solid-state detector (Piranha, RTI) was inserted at the center of the phantom and was scanned to obtain a dose rate profile (mGy/s) [14, 15]. Volume scanning was performed at tube voltages of 80 and 100 kV, a 320-mm-diameter effective field-of-view, rotation speed of 500 ms/rot, slice width of 0.5 mm × 320, and 160-mm scan range (160-mm width acquisition in a single non-helical rotation). For OEM_OFF,_ the tube current was set to “SD (standard deviation of image noise).” Table 1 shows the tube currents observed (values displayed on system console) after changing to OEM_ON_ and the resulting decrease in X-ray output.

**Table 1:** Changes in tube current between OEM_OFF_ and OEM_ON_

Tube current^a^ (mA)	500	400	300	250	200	150	130	100	90	80	70	60	50	40
SD setting at 80 kV^b^	14.5	16.5	19.5	21.7	25.2	30.0	32.5	37.0	40.0	43.0	45.0	50.0	55.0	63.0
SD setting at 100 kV^b^	8.7	9.8	11.5	12.6	14.4	16.5	18.0	21.0	22.0	23.0	25.0	27.0	30.0	34.0
Tube current at OEM_ON_^c^ (mA)	425	340	255	213	170	133	118	95	88	80	70	60	50	40

Tube voltage: 80 kV and 100 kV, X-ray tube rotation speed: 0.5 s/rot, slice thickness: 0.5 mm, slice width: 160 mm, effective field-of-view: 320 mm

^a^OEM_OFF_ tube current

^b^SD setting required to adjust output at a given tube current

^c^Tube current displayed on the control console during OEM_ON_ relative to the tube current for OEM_OFF_ as determined by the standard deviation of image noise (SD) setting at each tube voltage

#### X-ray output modulation angle

The X-ray output modulation angle was calculated using Eq. 1 assuming a single rotation of a volume scan and modulation through 360° at 0.5 s/rot, where θ is the X-ray output modulation angle (°), T_1rot_ is the time taken for a single rotation, and T_drr_ is the dose rate reduction time (ms). T_drr_ was obtained by comparing the dose rate profiles of OEM_OFF_ and OEM_ON_. However, due to variations at each of the plotted points forming the dose rate profiles, the start and finish times of dose rate reduction could not be measured accurately. For this reason, peaks before and after the start of dose rate reduction were identified, and the start and finish times were recorded when 1% modulation was observed relative to the peak value. In addition, the results for tube currents of 100, 90, and 80 mA were excluded for θ evaluation.

1 $$\theta = 360 \times \frac{1}{{T_{{1{\text{rot}}}} }} \times {\text{T}}_{drr}$$

#### Maximum X-ray output modulation rate (DM_Max_) and total X-ray output modulation rate (DM_Total_)

X-ray output modulation rate (DM) was calculated according to the dose rate profiles for OEM_OFF_ and OEM_ON_ using Eqs. 2 and 3:

2 $$DM_{Max} = \left[ {1 - \frac{{DR_{{OEM_{ON} }}^{Max} }}{{DR_{{OEM_{OFF} }} }}} \right] \times 100$$

3 $$DM_{Total} = \left[ {1 - \frac{{D_{{OEM_{ON} }}^{Total} }}{{D_{{OEM_{OFF} }}^{Total} }}} \right] \times 100$$

where DM_Max_ is the maximum X-ray output modulation rate (%), DR_Max_OEM_ON_ is the dose rate with the greatest reduction after changing to OEM_ON_, DR_OEMOFF_ is the dose rate obtained with OEM_OFF_, DM_Total_ is the total X-ray output modulation rate (%), and $D_{{OEM_{ON} }}^{Total}$ and $D_{{OEM_{OFF} }}^{Total}$ are the total doses (mGy) during OEM_ON_ and OEM_OFF_, respectively.

#### Image noise

**Fig. 1:**
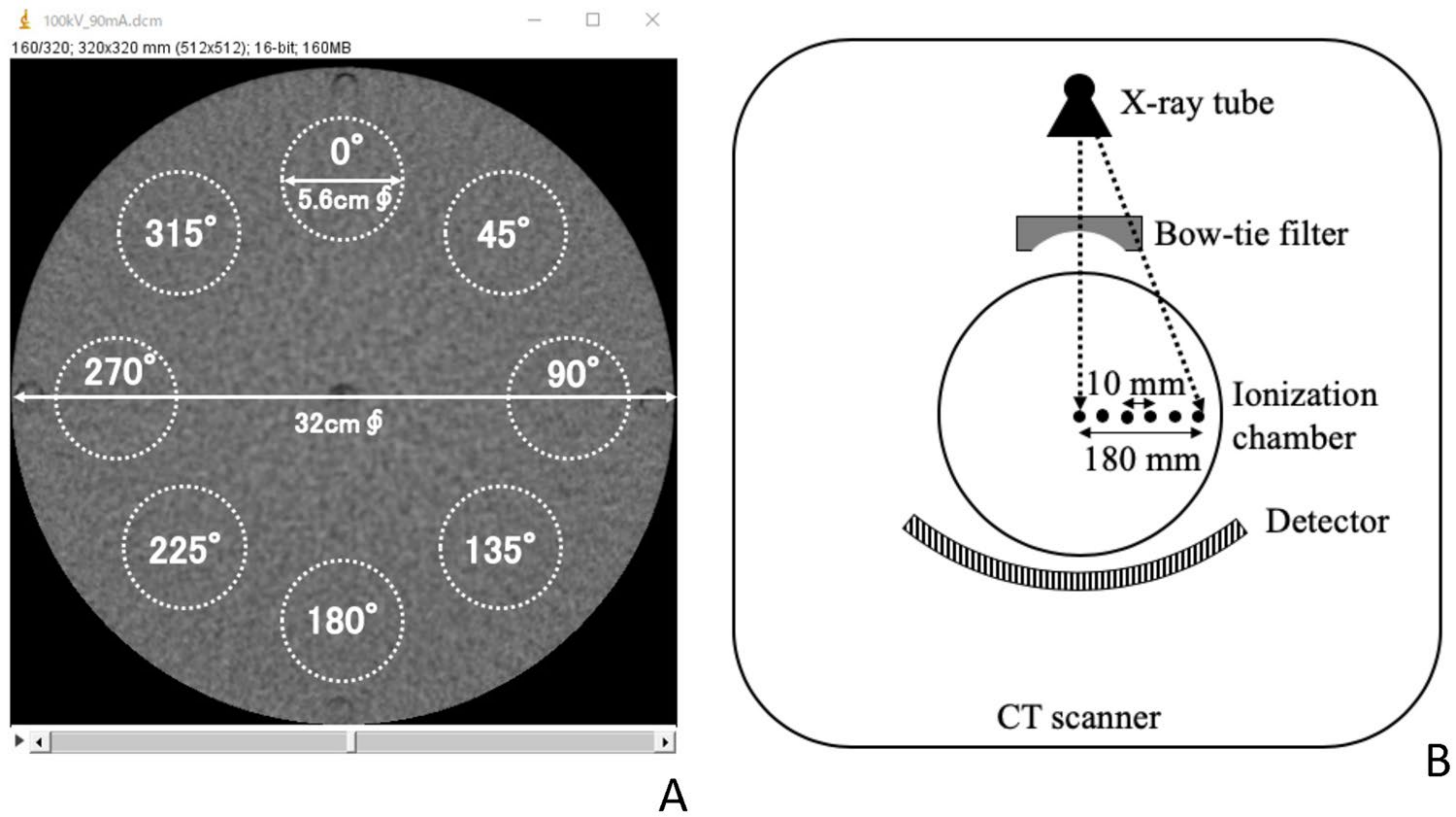
Geometric arrangement used in experiments. **a** Measurement of image noise: Images were reconstructed with 0.5-mm slice thickness and 0.5-mm slice width; the 5 middle slices were chosen; 8 regions of interest (5.6 cm diameter) were positioned on each slide image; and image noise (SD_ON/OFF_) was measured in those regions. **b** Bowtie filter shape evaluation: An ionization chamber was moved in 10-mm increments up to 180 mm from the isocenter along the x-axis and air kerma was measured at each step. To perform these measurements, the X-ray tube needed to be fixed in the 12 o’clock direction using “service mode”

The solid-state detector was removed from the center of the PMMA phantom and replaced with a 1-cm-diameter PMMA cylinder. After scanning under the conditions described above, images were reconstructed with a slice thickness of 0.5 mm and a slice width of 0.5 mm, after which image noise (SD_ON/OFF_) was measured in 8 prescribed regions of interest (each 5.6 cm in diameter) per slice in 5 axial slices taken from the center of the reconstructed image, thereby yielding the percentage change in noise (n) (Fig. 1a).

4 $$n = \left[ {\frac{{SD_{{OEM_{ON} }} }}{{SD_{{OEM_{OFF} }} }} - 1} \right] \times 100$$

### Evaluated subjects

Exposure dose was evaluated in 8 participants who underwent a fetal CT scan on the same CT system between 2010 and 2017 (3 cases of short extremities, 1 case of achondrogenesis, 1 case of thanatophoric dysplasia, 2 cases of other fetal disorders, and 1 case of threatened premature labor); the expectant mothers were aged 30 ± 6 years, had an abdominal circumference of 95 ± 7.7 cm, were weighing 62 ± 4.7 kg, and were at 32 ± 2 gestational weeks.

### ImPACT MC software

ImPACT MC Version 1.60 (CT imaging GmbH, Erlangen, Germany) MC simulation software is capable of evaluating, based on photon interactions, the absorbed dose per individual voxel in DICOM images obtained by CT. The software program can process data such as bowtie filters, air kerma, energy spectra, CT-to-density conversion factor, and scanning conditions [16, 17]. The data needed to run the MC simulation were measured based on the tube voltage, effective field of view, and other conditions used in the fetal CT scans.

#### Measuring bowtie filter shape

A free-in-air pencil ionization chamber (10X-3CT, Radcal Corp., Monrovia, CA, USA) was positioned at the isocenter of the CT system. The ionization chamber was moved in 10-mm increments up to 180 mm from the isocenter along the x-axis direction, and air kerma was measured at each step using an ionization chamber dosimeter (Model 9015, Radcal Corp., Monrovia, CA, USA) (Fig. 1b). To perform these measurements, the X-ray tube had to be fixed in the 12 o’clock position using “service mode.” The percentage reduction in air kerma observed in these measurements was then used as the virtual bowtie filter in MC simulations.

#### Measurement of air kerma and half-value layer and estimation of energy spectra

After positioning the free-in-air pencil ionization chamber at the isocenter of the CT system, air kerma was measured per scan rotation using an ionization chamber dosimeter (accuracy: ± 4%). Next, the solid-state detector (Black Piranha, RTI group AB, Mölndal, Sweden; 133 mm H × 75 mm W × 26 mm D) was placed on the system bed and the half-value layer was measured (accuracy: ± 10% or 2 mm) [18]. X-rays oblique to the orientation of rotation that fell outside a 10-mm length detector area were screened by covering with a 3-mm thick lead (99.9%) case (Acrobio Corp., Tokyo, Japan; 147 mm H × 105 mm W × 58 mm D). Energy spectra corresponding to the measured half-value layer were then estimated using the ImPACT MC spectra creation tool (based on the estimation method used by Tucker et al. [19]).

#### CT value-to-density conversion

Although the DICOM images obtained from CT scans contain CT values, the MC method requires a density value for each voxel. CT values were converted to density values using a standard conversion graph in the software program that was created from the CT values of water (0 HU: 1 g/cm^2^) and air (− 1000 HU: 0 g/cm^2^).

#### MC simulation conditions

Table 2 shows the scanning conditions used in the actual clinical fetal CT scans with OEM_OFF_. Next, the DM for OEM_ON_ was estimated by linear approximation based on the relationships among the basic characteristics of OEM (Table 1). The tube current during OEM_ON,front_, which reduced the absorbed dose on the anterior side, and OEM_ON,back_, which reduced the absorbed dose on the posterior side, modulated DM_Max_ and DM_Total_ at the obtained θ values (see Sect. 3.1.1). MC simulations were then run with these scanning conditions; the mean dose absorbed by the organs and tissues (D_T,R_) was measured based on the resulting absorbed dose distribution, and the effective dose (ED) was calculated.

**Table 2:** MC simulation conditions based on imaging conditions used in clinical examinations

No.	Tube voltage (kV)	Scan	Slice width (mm)	Field of view (mm)	Tube current 1 OEM_OFF_/OEM_ON_^a^ (mA)	Tube current 2 OEM_OFF_/OEM_ON_^a^ (mA)	Tube current 3 OEM_OFF_/OEM_ON_^a^ (mA)	Scan rotation time (sec/rot)	Air kerma^b^ (mGy/100 mAs)
A	80	3	160	320	220/187	220/187	220/187	0.5	11.17
B	80	3	160	320	172/149	217/185	202/172	0.5	11.17
C	80	3	140	320	157/138	217/185	232/198	0.5	11.17
D	100	3	120	360	110/103	120/110	110/103	0.5	19.11
E	100	3	120	320	100/95	110/103	105/99	0.5	19.08
F	100	3	128	320	115/107	145/129	125/114	0.5	19.08
G	100	2	160	320	85/84	105/99		0.5	19.08
H	100	3	140	320	80/80	90/88	85/84	0.5	19.08

^a^OEM_ON_ tube current was estimated by linear approximation from the results in Table 1

^b^Air kerma data are actual measurements

### Evaluating exposure dose

#### Mean dose absorbed by organs and tissues (D_T,R_)

D_T,R_ (mGy) measurements were obtained by choosing around 3 regions of interest in each organ in DICOM images, depending on organ size. To determine full-body exposure to the fetus, where possible, we obtained measurements from the organs and tissues required to evaluate the ED. However, when an organ could not be discerned due to the low contrast of the DICOM images and small organ size, regions of interest were positioned based on anatomical relationships. The D_T,R_ of red bone marrow and mineralized bone was calculated using the evaluation method described by Nishizawa et al. [20] and reference data (Table 4.2 and Table A 1) [21] shown in ICRP Publication 110 used in constructing a reference person. As the pregnant woman receives exposure to only the abdominal region, five abdominal organs were selected for measurement (liver, kidneys, colon, uterus, and bladder). However, when any of these organs were outside the scanned area, the organ was excluded from evaluation.

#### Effective dose (ED)

ICRP Publication 103 [22] defines ED as a value calculated for an adult reference person and that ED is unrelated to body type, age, or sex. ICRP Publication 103 also mentions that ED should not be used to evaluate exposure dose to individuals. Given that this study specifically evaluates pregnant women and fetuses and verifies the effects of OEM per patient, we chose to evaluate the effect of OEM using ED(Sv), a parameter that goes beyond the scope of the prescribed definition of ED.

5 $$ED = \mathop \sum \limits_{T} W_{T} \mathop \sum \limits_{R} W_{R} D_{T,R}$$

where W_T_ is the tissue weighting factor (ICRP Publication 103 [22]) and W_R_ is the radiation weighting coefficient (1.0). We verified the effect of OEM on fetal CT scans based on the above parameters. This has been approved by the Institutional Review Board (HM18-433).

## Results

### Basic characteristics of OEM

#### X-ray output modulation angle (θ)

**Fig. 2:**
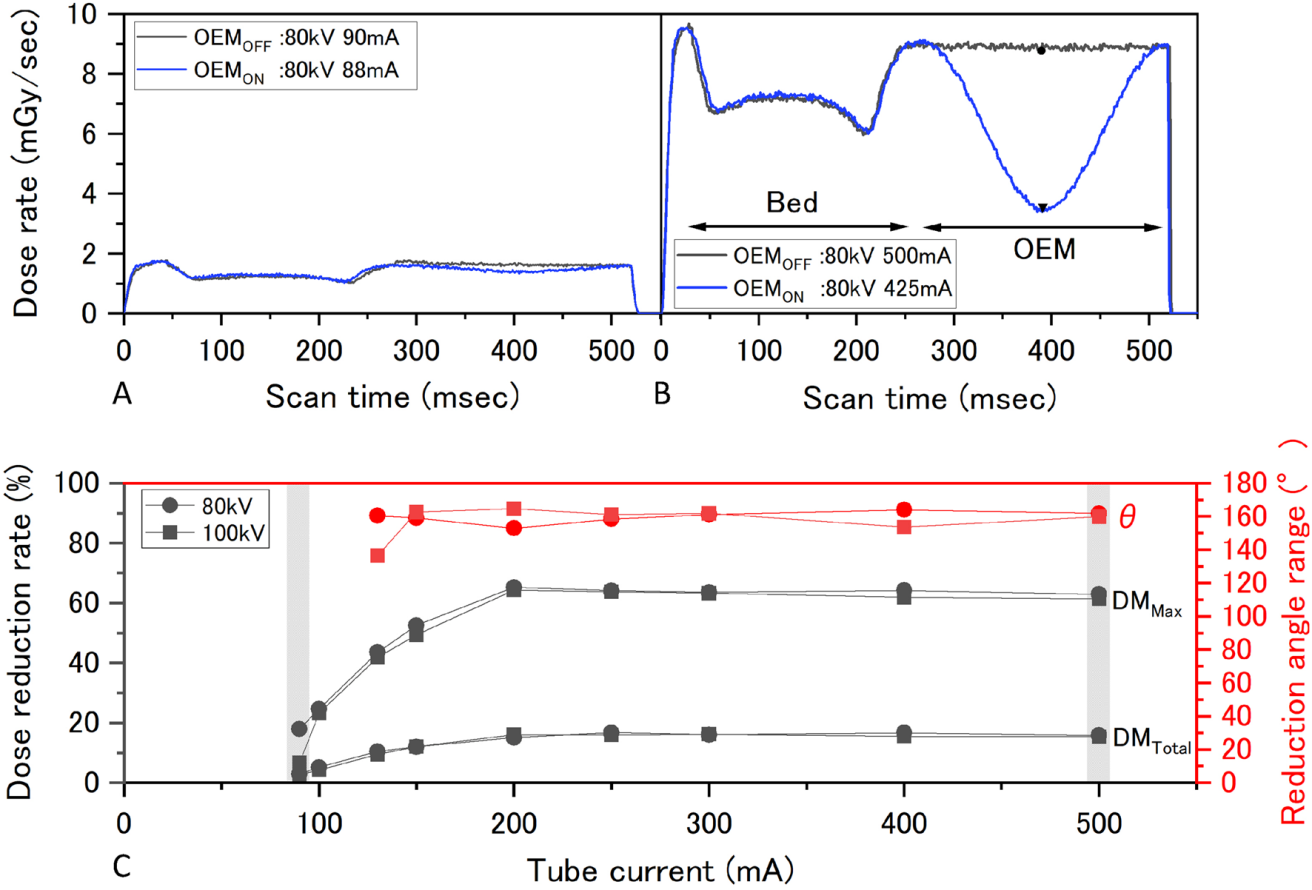
Evaluating basic characteristics of OEM. **a** Dose rate profile at 80-kV tube voltage and 90 mA tube current (OEM_ON_: 88 mA) for OEM_OFF_ showing modulation of a small proportion of dose rate. **b** Dose rate profile at 80-kV tube voltage and 500 mA tube current (OEM_ON_: 425 mA) for OEM_OFF_ showing modulation of a markedly large proportion of dose rate. **c** Change in X-ray output modulation angle (θ), maximum X-ray output modulation rate (DM_Max_), and total X-ray output modulation rate (DM_Total_) relative to tube current. The gray areas show the results from scanning conditions used in A and B, respectively

An X-ray tube rotation speed of 500 ms/rot shows that a single rotation requires 500 ms. The OEM_OFF_ and OEM_ON_ dose rate profiles were measured at this rotation speed. Figure 2a and b shows the dose rate profile for OEM_OFF_ at an 80-kV tube voltage and 90-mA tube current (OEM_ON_: 88 mA), showing modulation at a small proportion of dose rate, and the dose rate profile for OEM_OFF_ at an 80-kV tube voltage and 500-mA tube current (OEM_ON_: 425 mA), showing modulation at a markedly large proportion of dose rate. Based on the dose rate profiles, the X-ray output time from start-up to shutdown was 527.8 ± 4.0 ms. Assuming a rotation speed of 525 ms, the X-ray tube rotated through 0.686° every 1 ms (360°/525 ms) and, therefore, OEM_ON_ θ = 160.1 ± 3.5°. At 100 kV and 130 mA, the angle θ was 136.5°, which was much smaller than all other angles and was excluded from evaluation as an outlier.

#### Maximum X-ray output modulation rate (DM_Max_) and total X-ray output modulation rate (DM_Total_)

DM_Max_ showed a dose reduction of 63.5% ± 1.1% at 200 mA and higher, independent of tube voltage. Similarly, DM_Total_ showed a dose reduction of 16.0% ± 0.5% at 200 mA and higher, independent of tube voltage. In addition, both DM_Max_ and DM_Total_ gradually reduced in size below 200 mA, and modulation was absent below 80 mA. DM data taken from dose rate profiles for 90 mA and 500 mA tube currents are highlighted in gray in Fig. 2c. Dose rate profiles also allowed us to verify the effect of X-ray absorption by the bed.

#### Image noise

**Fig. 3:**
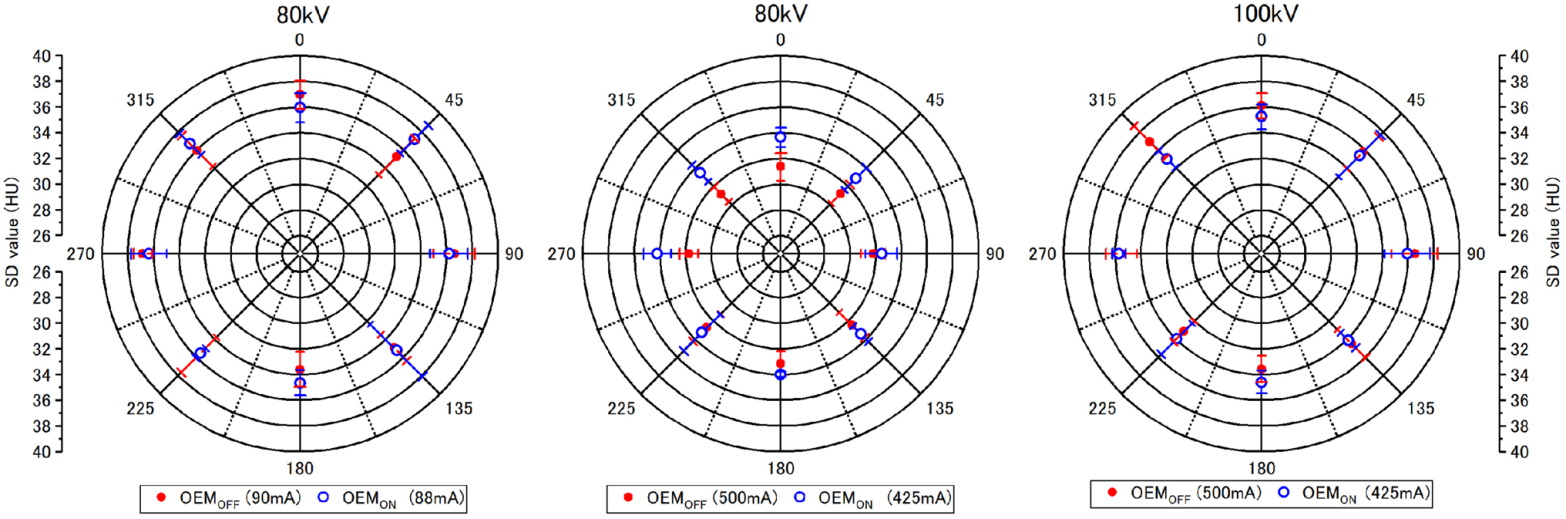
OEM effect on image noise. The effect of OEM on image noise was evaluated in terms of changes in tube current and tube voltage

Figure 3 shows SD plotted on polar coordinates during OEM_ON_ and OEM_OFF_ for three representative scanning conditions. These three scanning conditions show characteristic image noise measurements obtained from the phantom. Considering the change in noise evaluated based on SD, there was no difference in n between OEM_ON_ and OEM_OFF_ in any angular direction for an 80 kV tube with a current of 90 mA that exhibits low DM. At a tube current of 500 mA, in the 0° ± 90° range where there were concerns over the effect of OEM, we observed an increase of 6.0% ± 2.4%. Similarly, the change in useful X-ray output in fetal CT scans was 3.2% ± 3.3% at 400 mA, 2.4% ± 1.0% at 300 mA, and − 0.5% ± 2.5% at 250 mA, showing that the smaller the DM, the smaller the effect on n. In contrast, in the 180° ± 90° range, the change was 3.4% ± 2.5% at 500 mA, 1.9% ± 1.1% at 400 mA, and 1.4% ± 2.0% and 0.3% ± 0.8% at 300 and 250 mA, respectively, showing a smaller effect on n compared to that of the 0° ± 90° range. The greatest effect on n was 7.8% at 500 mA in the 270° direction. For a tube voltage of 100 kV, at a tube current of 500 mA, the effect on n in the 0° ± 90° range was − 1.9% ± 2.1% and that in the 180° ± 90° range was 0.7% ± 2.0%, indicating no effect of OEM and implying a difference caused by tube voltage (Fig. 3).

### ImPACT MC

#### Bowtie filter shape

**Fig. 4:**
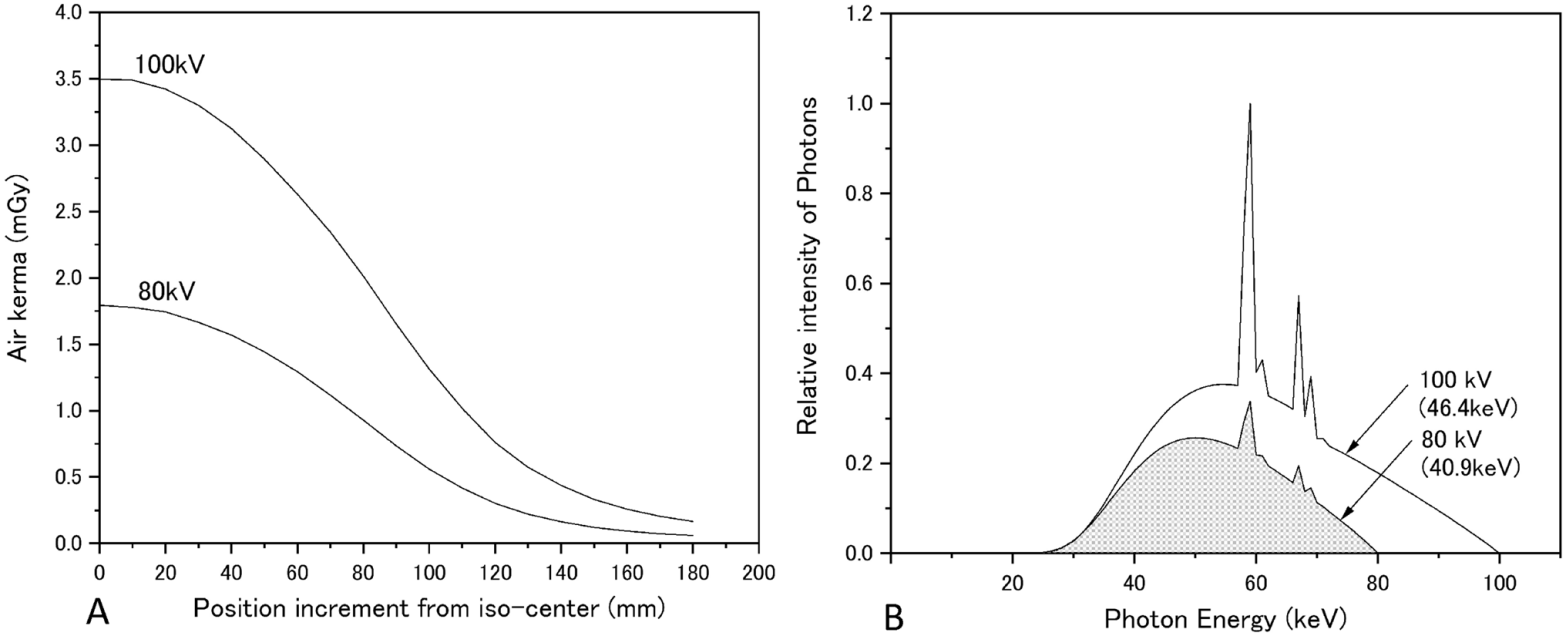
Basic data used in MC simulations. **a** Bowtie shapes. **b** Energy spectra

Air kerma, which was measured in the x-axis direction, became smaller the further it was measured from the isocenter (Fig. 4a). A bowtie filter that produces this reduction in air kerma is bilaterally symmetrical around the central axis of the isocenter (0 mm); hence, the CT system used in MC simulations was equipped with a bowtie filter.

#### Air kerma, half-value layer, and energy spectra

For the 320-mm effective field-of-view commonly used in CT scans, at a tube voltage of 80 kV, the air kerma was 11.17 mGy/100 mAs, and at 100 kV, the air kerma was 19.08 mGy/100 mAs (Table 2). Figure 4b shows energy spectra that correspond to half-value layers of 4.76 mmAl (40.9 keV) and 6.03 mmAl (46.4 keV) for each tube voltage and were measured based on a 320-mm effective field-of-view size. The energy spectra show tungsten-specific X-rays with values of 59 keV, 61 keV, 67 keV, and 69 keV (Kα1: 59.32 keV, Kα2: 57.98 keV, Kβ1: 67.24 keV, and Kβ2: 67.42 keV, respectively).

### Evaluating exposure dose

**Fig. 5:**
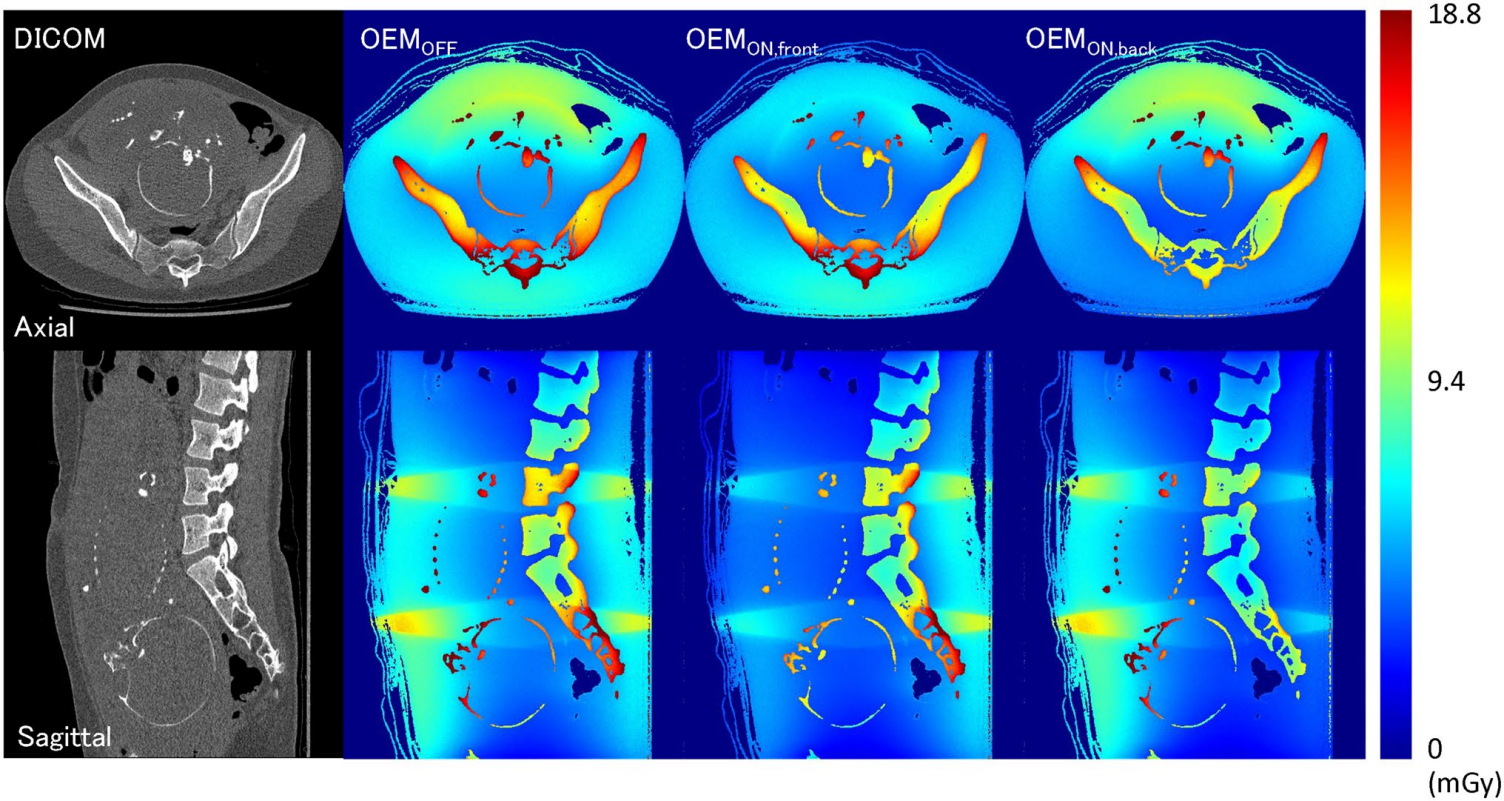
Absorbed dose profiles obtained by MS simulation

**Fig. 6:**
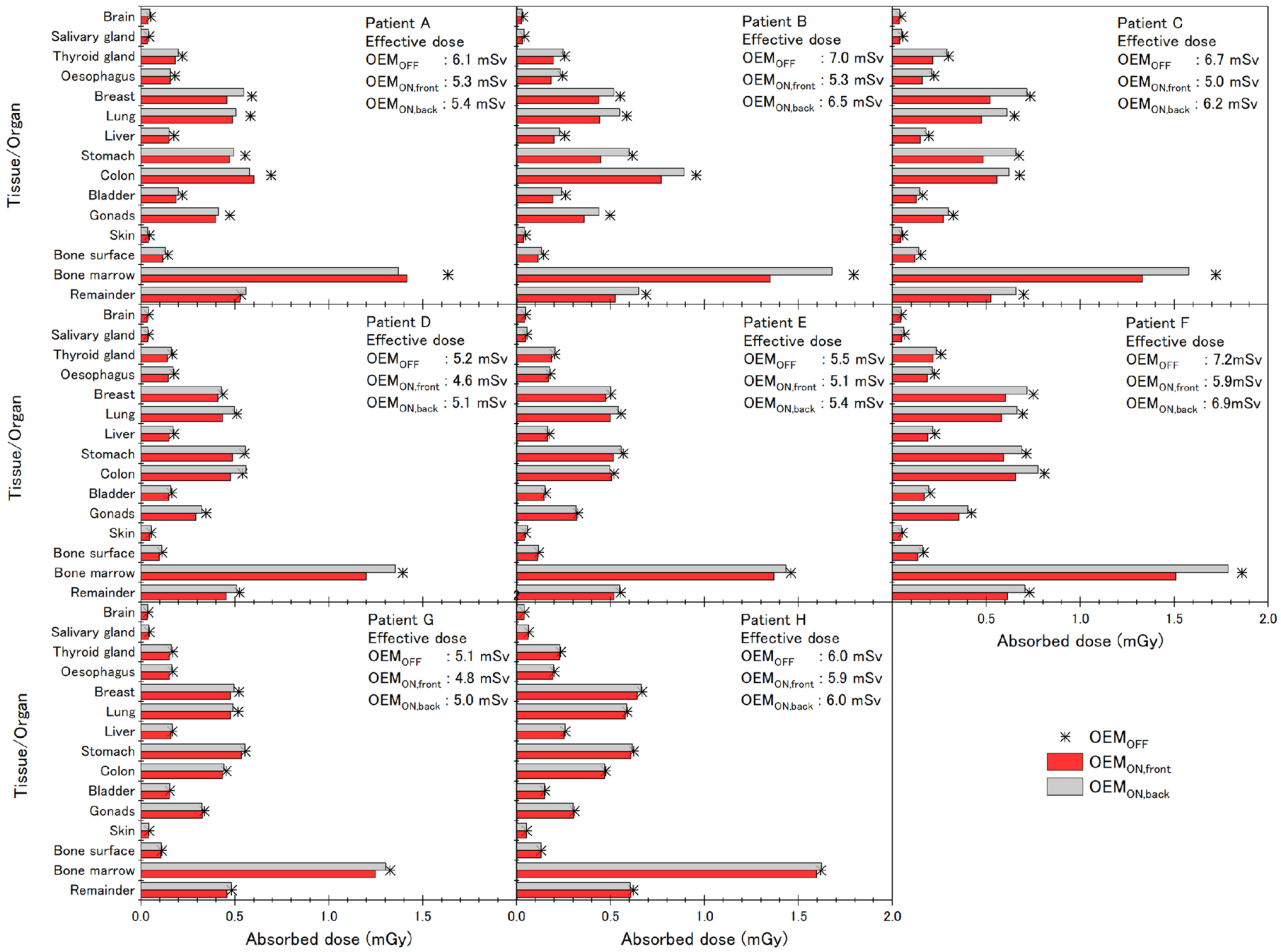
Comparison of dose absorbed by fetus organs and tissues (D_T,R_) and effective dose (ED). Fetal CT scans of patients A–H simulated using the scanning conditions shown in Table 2

OEM scan conditions (θ = 160°) that produced a large DM in fetal CT scans were OEM_ON,front_ and OEM_ON,back_ (Figs. 5, 6). The results indicated that OEM produced a greater reduction in D_T,R_ in the thoraco-abdominal region than in the head and neck region, although OEM did not reduce D_T,R_ for specific organs in the thoraco-abdominal region. Further, D_redmarrow_ was the highest absorbed dose in all fetuses. For D_T,R_ and ED where DM was large at a tube voltage of 80 kV, the dose to the fetuses scanned at this voltage (patients A–C, OEM_OFF_: 6.6 ± 0.4 mSv, OEM_ON,front_: 5.2 ± 0.1 mSv) was reduced by 1.4 mSv (27%), which was larger than the 0.5 mSv (9%) reduction observed at 100 kV (patients D–H, OEM_OFF_: 5.8 ± 0.9 mSv, OEM_ON,front_: 5.3 ± 0.6 mSv). A dose reduction was also observed with OEM_ON,back_ (patients A–C: 6.1 ± 0.6 mSv [8%], patients D–H: 5.7 ± 0.8 mSv [2%]), but the reduction was not as large, as the OEM_ON,front_ and D_T,R_ reductions were absent in specific organs.

**Fig. 7:**
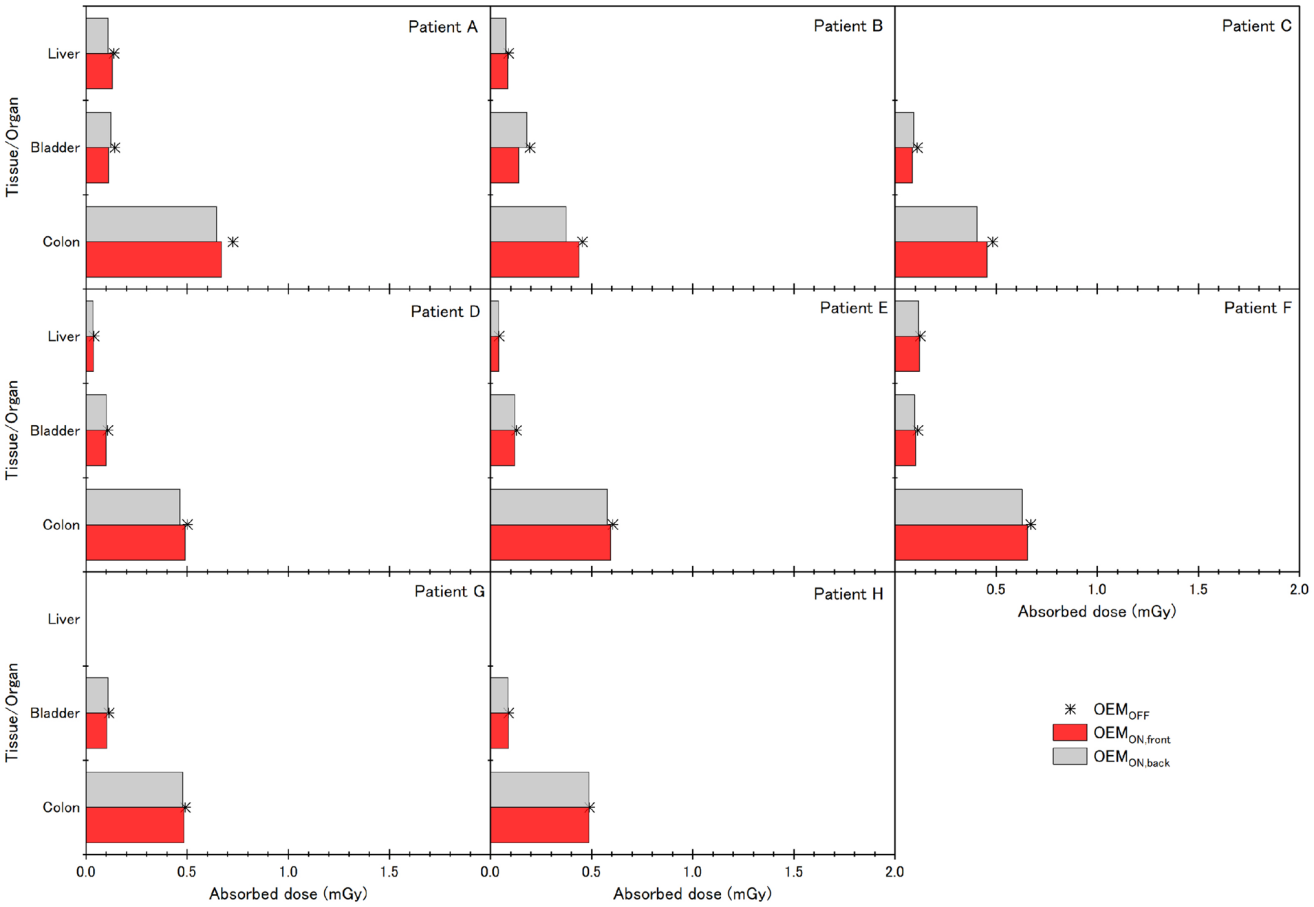
Comparison of doses absorbed by organs and tissues of pregnant women (D_T,R_). Fetal CT scans of patients A–H simulated using the scanning conditions shown in Table 2. In patients C, G, and H, the liver of the pregnant woman was outside the scanning range and did not appear in the DICOM image; hence, dose values for the liver are not shown

For pregnant women, D_T,R_ was evaluated in the liver, bladder, and colon when these organs appeared in DICOM images (Fig. 7). D_colon_ for OEM_OFF_ was 0.55 ± 0.10 mGy, that for OEM_ON,front_ was 0.53 ± 0.09 mGy (4%), and that for OEM_ON,back_ was 0.51 ± 0.10 mGy (8%), showing a very slightly larger dose reduction with OEM_ON,back_. These results indicated that the dose reduction achieved using OEM was not as large in pregnant women as in fetuses.

## Discussion

We measured the basic characteristics of OEM and ran MC simulations with these data to evaluate the effect of OEM on fetal CT scans in terms of image quality and exposure dose to the fetus and pregnant woman. This evaluation indicated that OEM was effective at reducing the exposure dose to the fetus but did not have a substantial effect on the exposure dose to pregnant women. Accordingly, we intend to actively implement dose reduction only for the fetus by modulating X-ray output with OEM_ON,front_. Given that DM increases at higher tube currents, DM is absent at 80 mA or below, and that n is higher in the 0° ± 90° range at a tube voltage of 80 kV but this difference is absent at 100 kV, these tube current characteristics and image quality characteristics must be considered when setting scanning conditions for OEM_ON,front_.

Measurements revealed that the T_1rot_ of the CT system was approximately 25 ms longer than the 500-ms set value. Part of this extra time is needed for the X-ray output to stabilize (start-up) and is handled as exposure not used for image reconstruction, and it results in a region of overlapping X-ray output. There are no findings that support attributing this region solely to start-up time, and the possibility cannot be ruled out that short periods of overlap will continue to occur after stabilization of X-ray output. To evaluate θ, T_1rot_ was taken as the time from start-up to shutdown. The θ = 160° finding was obtained by using T_1rot_ = 500 ms and did not include X-ray tube rotation time characteristics. Shorter rotation times are expected to make control of θ more difficult; thus, using shorter rotation times will require separate evaluation of θ. As such, the X-ray output start-up cannot be easily recreated by an MC method; hence, it was not incorporated into the MC simulations. Moreover, θ decreased to 136.5° at a tube voltage of 100 kV and a tube current of 130 mA. This reduction was caused by measurements taken from a dose rate profile of a scan that ended while DM was still ongoing. Normally, when scanning is performed without orbital synchronization, the X-ray tube starts scanning from a random angle. Depending on the X-ray tube angle at which scanning begins, the scan may end while X-ray output modulation is ongoing, or the X-ray output may not be modulated. This is not a major problem for helical scans, as DM is not implemented during the first rotation and X-ray output is modulated only in the appropriate angular range during subsequent successive rotations [23]. However, volume scanning acquires a wide slice thickness in only a single rotation; thus, any issue that prevents X-ray output modulation during this rotation is unacceptable. This also applies to wide-volume scanning that involves consecutive volume scans.

OEM started modulating X-ray output at tube currents of 90 mA and above independent of tube voltage. The modulation rate in terms of DM_Max_ and DM_Total_ was also constant at tube currents of 200 mA and above. Accordingly, OEM is not expected to be effective at low tube currents, below 90 mA, but at tube currents of 200 mA and higher, the behavior mentioned above will produce a dose-reducing effect. Given these findings, when the objective is to reduce the exposure dose, we propose selecting a low tube voltage in the scan settings to obtain high tube currents.

In contrast, when OEM was used at high tube currents, the results indicated that n increases in the 0° ± 90° range together with substantial DM. When the objective is to avoid reducing image quality due to image noise, when the tube voltage is 80 kV, we propose selecting a current of 250 mA or below or selecting a tube voltage of 100 kV in the settings. When the objective is to both reduce exposure dose and ensure image quality, we propose scan conditions of 80 kV at 200–250 mA or 100 kV. A tube voltage of 100 kV also resulted in lower contrast images than those obtained with 80 kV. When the objective is only a morphological diagnosis such as in cases of osteogenesis imperfecta, though increasing the tube voltage may prevent any loss of diagnostic performance, it also causes an unavoidable increase in exposure dose; hence, both diagnostic performance and exposure must be considered in such cases. Ultimately, each facility makes their own decisions on scanning conditions and use the various findings collected in this article as a reference.

Running an MC simulation of a CT scan requires a bowtie filter and energy spectra. We estimated the bowtie filter shape (Fig. 4a) based on X-ray transmission using the measurement method recommended by ImPACT MC software. The energy spectra estimated from the measured half-value layer (Fig. 4b) showed an absorption edge at an energy close to that of the K-absorption edge [24, 25], which arises from estimating primary X-ray energy spectra using Klein–Nishina differential cross sections based on actual measurements of Compton scattered photons from the CT system that are collected with a cadmium telluride detector. Accordingly, MC simulations were also run on these energy spectra.

The exposure dose in normal fetal CT scans (OEM_OFF_) was highest on the anterior side (of the pregnant woman), followed by the posterior side, and then the lateral side (Figs. 5, 6). This difference in exposure dose is attributed to X-ray absorption by the bed and differences in body thickness. OEM_ON,front_ also reduced the dose absorbed by the anterior side and OEM_ON,back_ reduced the dose absorbed by the posterior side (Figs. 5, 6). In both these situations, we had difficulty reducing the dose absorbed in the center of the axial section of the pregnant woman. In utero, the fetus is positioned with its head in the pelvic cavity (toward the center of the axial section) and its thoraco-abdominal region at the body surface pushing against the lower abdominal region of the pregnant woman. Consequently, OEM causes a greater reduction in D_T,R_ in the thoraco-abdominal region of the fetus than in the head and neck region (Figs. 5, 6). Given that D_T,R_ is affected by both the position of the fetus and the overlapping of X-rays that occurs due to slice width and the number of slices along the body axis, OEM will not reduce the dose to specific organs. Furthermore, for all fetuses, the findings indicated that D_T,R_ tended to be highest in the breasts, lungs, stomach, colon, gonads, and red bone marrow. The highest of these was D_red marrow_, which was calculated as a weighted value according to the weight distribution of the red bone marrow and based on the evaluation method proposed by Nishizawa et al. [20]. Although D_red marrow_ was the highest (Patient F, OEM_OFF_: 1.86 mGy), the D_T,R_ during fetal CT scans was still acceptably lower than the dose received during abdominal CT scans (mean: 8 mGy, maximum: 49 mGy) or pelvic CT scans (mean: 25 mGy, maximum: 79 mGy) performed for image-based diagnosis of the pregnant mother [6]. If the fetal dose is evaluated on the basis of the D_red marrow_, then detailed informed consent will be needed for the CT scan, as the fetal dose exceeds 1 mGy. However, as the fetal dose is 100 mGy or less, the increased risk of prenatal death, deformity, or mental retardation is almost never large enough to be detectable above spontaneous incidence [6]. Methods for evaluating this type of exposure dose to the fetus and organs proposed thus far have used fundamental experiments in human phantoms [26, 27], MC methods in reference human digital phantoms [28], or simplified dose estimation methods [10, 29, 30]. In this study, fetus D_T,R_ was estimated in detail by MC simulation based on clinical images, and ED was calculated.

The ED to the fetus associated with a fetal CT scan was determined to be 5.1–7.2 mSv. However, because the purpose of this study was to examine the effect of OEM on fetal CT scans, clinical cases that used scanning conditions with an unmodified X-ray output were excluded from the study. Among the cases we evaluated, OEM_ON,front_ was extremely effective in Patients B and C (tube voltage, 80 kV; maximum tube current, 232 mA), as there was only minor reduction in image quality in the 0° ± 90° range. Fetal CT scans also cause unnecessary medical exposure to the pregnant woman. For this reason, we evaluated OEM_ON,back_ as a means of maximally reducing the exposure dose to the pregnant woman. However, as there was only a limited reduction in D_T,R_ to the liver, bladder, and colon of the pregnant woman with OEM_ON,back_ (Fig. 7), it would seem more advantageous to pursue obtaining advanced informed consent that allows preferential use of OEM_ON,front_.

## Conclusion

In this paper, we evaluated the utility of OEM in fetal CT scans from the standpoint of image quality and exposure dose to the pregnant mother and fetus. With proper understanding of the characteristics of OEM, it can serve as an effective tool for dose reduction in fetal CT scans. Similar to using OEM for exposure dose reduction to breasts and the lens of the eye, OEM should also be encouraged for fetal CT scans.

## Data Availability

ImPACT MC Version 1.60 (CT imaging GmbH, Erlangen, Germany) MC simulation software.

## Appendix

6 $$D_{red marrow} = \sum md \times \frac{1}{M}$$

where m is the weight of the red bone marrow being measured, d is the dose measured in the red bone marrow, and M is the weight of all red bone marrow in the body [20]. Mineralized bone was also calculated with Eq. 6 using an exposure dose in the same locations.
